# Drone-Borne Magnetic Gradiometry in Archaeological Applications

**DOI:** 10.3390/s24134270

**Published:** 2024-07-01

**Authors:** Filippo Accomando, Giovanni Florio

**Affiliations:** Department of Earth, Environmental and Resources Sciences, University of Naples “Federico II”, 80126 Naples, Italy; filippo.accomando@unina.it

**Keywords:** UAV magnetic survey, vertical gradient configuration, archaeological prospections

## Abstract

The use of magnetometers arranged in a gradiometer configuration offers a practical and widely used solution, particularly in archaeological applications where the sources of interest are generally shallow. Since magnetic anomalies due to archaeological remains often have low amplitudes, highly sensitive magnetic sensors are kept very close to the ground to reveal buried structures. However, the deployment of Unmanned Aerial Vehicles (UAVs) is increasingly becoming a reliable and valuable tool for the acquisition of magnetic data, providing uniform coverage of large areas and access to even very steep terrain, saving time and reducing risks. However, the application of a vertical gradiometer for drone-borne measurements is still challenging due to the instability of the system drone magnetometer in flight and noise issues due to the magnetic interference of the mobile platform or related to the oscillation of the suspended sensors. We present the implementation of a magnetic vertical gradiometer UAV system and its use in an archaeological area of Southern Italy. To reduce the magnetic and electromagnetic noise caused by the aircraft, the magnetometer was suspended 3m below the drone using ropes. A Continuous Wavelet Transform analysis of data collected in controlled tests confirmed that several characteristic power spectrum peaks occur at frequencies compatible with the magnetometer oscillations. This noise was then eliminated with a properly designed low-pass filter. The resulting drone-borne vertical gradient data compare very well with ground-based magnetic measurements collected in the same area and taken as a control dataset.

## 1. Introduction

Magnetic investigations are widely used for archaeological applications because magnetic anomalies are sensitive not only to the physical properties of natural sources (i.e., soils and rocks) but also to man-made objects, as in the case of the archaeological targets. Many diverse active or passive geophysical methods are used in archaeological investigations. Among the active geophysical methods, electric and electromagnetic surveys are the most useful (e.g., [1]), while among the passive methods, the magnetic method is widely used because it is effective, fast, cheap, and non-invasive (e.g., [2,3]).

However, archaeological areas are sometimes difficult or impossible to access (i.e., dense vegetation areas, swamps, steep mountain slopes, cultivated areas, lakes, glaciers, or foreshore areas), and ground magnetometric prospection may be unfeasible. To overcome these problems, in the last decade, the use of Unmanned Aircraft Systems (UAVs) combined with new miniaturized magnetometer sensors has become an attractive option in several situations. Drone-borne magnetic systems allow covering large areas flying at low altitudes, producing high-resolution datasets. UAV magnetic surveys have been realized for many applications, such as mineral exploration (e.g., [4,5,6,7,8,9,10]) or UXO (unexploded ordnance) detection (e.g., [11]). In engineering applications, drone magnetometry is used for the decommissioning of oil industry infrastructure or for the detection of buried metal pipelines (e.g., [12,13,14]). Drone magnetometry has also been used in geological applications for exploration of the subsurface ([15,16]) or volcano monitoring and geohazard assessment ([17]). Only a few examples of UAV magnetic surveys for archaeological targets have been attempted in the last years ([18,19,20,21]), maybe because of the low amplitude of the magnetic field anomalies generated from archaeological features and the required high-resolution grid of measurements needed in this field of exploration.

Usually the magnetic anomalies generated by archaeological targets are weak, spread over small areas and often interfere with each other. For these reasons, their identification requires high-resolution magnetic data, i.e., with a small spacing between the survey lines, collected close to the ground. The survey area should be large enough to make the resulting anomaly informative, especially in the case of regular and elongated shapes of remains from buried buildings or roads. A common survey practice in archaeological applications is to use a magnetic gradiometer (e.g., [22,23]). With this approach, two measurements of the magnetic field are acquired at the same time through a pair of magnetic sensors spaced at a fixed distance, called ‘baseline’. The difference between the two measurements obtained from the sensors divided by their distance allows the computation of an approximation of the magnetic gradient along the direction of the baseline. Often, the two sensors are arranged in a vertical direction, allowing the approximate computation of the vertical gradient of the magnetic field. The magnetic gradients computed in this way are insensitive to the temporal variations in magnetic fields, and the regional fields generally become negligible ([24]) so that the use of a magnetic gradiometer makes the use of a fixed base station during magnetic surveys non-mandatory. Another important feature of magnetic gradient data is that the resolution (i.e., the ability to distinguish the anomalies generated by nearby sources) is improved with respect to the magnetic field data. Finally, the vertical gradient of the magnetic field is more sensitive to the presence of shallower sources than the magnetic field, since it decays faster. The choice of the baseline length should be smaller than the distance between the sensor closer to the ground and the source depth. In the case of deep targets, as in aeromagnetic applications, the distance between the sensors should be increased. Usually, the baseline for hand-held magnetometers ranges between a minimum of 0.25 m and a maximum of 1 m.

The vertical gradient of magnetic measurements could, of course, also be computed from single sensor data using the linear filter well-known in potential field theory (e.g., [25]). Some authors tried to obtain a vertical gradient in UAV surveys by using two sets of horizontally co-located measurements at different altitudes measured during repeated flights ([26]). While, in some cases, these strategies can lead to good results, the resulting gradients may still contain the effects of diurnal variations not properly compensated or of positioning errors (when using two datasets at different altitudes).

In this study, we will show the results of a first attempt to measure the drone-borne vertical gradient of the total field by arranging the magnetic sensors of the Geometrics Micro-Fabricated Atomic Magnetometer (MFAM) as a gradiometer. A special custom bird was developed to house the magnetometer, and an additional frame was used to separate the two sensors at a distance of 0.25 m. The results of a vertical gradient ground survey were used to validate the quality of the measurements collected with the drone.

## 2. Materials and Methods

In the archaeological site of Metaponto, in Southern Italy, ground and UAV surveys were made with the aim of testing, for the first time, a vertical gradient configuration with the MFAM. The intent was to explore the apsidal area of the Greek temple dedicated to Hera.

The sanctuary of Hera (so-called “Tavole Palatine”) extends 4 km away north-west of Metaponto (Southern Italy), around a monumental temple structure positioned inside a wall, which also encloses further small buildings and an altar. This region, a vital hub of Magna Graecia, is today the site of significant archaeological evidence. Located near the Bradano River, the Tavole Palatine were an integral part of a rural sanctuary. The first traces of cultural activity in the sacred area date back to the mid-7th century BC, as documented by the abundant archaeological material, such as archaic pottery and metal objects, including rings and examples of temple keys (e.g., [27]). The Doric temple of the goddess dates to the 6th century BC. Its dimensions are 16 m by 33 m, with a three-step crepidoma, six columns on the short sides, and twelve on the long ones, internally divided into a cell with a single nave (e.g., [28,29]). Ref. [30] described the main materials used for buildings and the quarry from where they were extracted. In the temple, a fine-grained yellow calcarenite was exclusively used, while rare blocks of the crepidoma are represented by a cemented conglomerate of fluvial origin.

**Figure 1:**
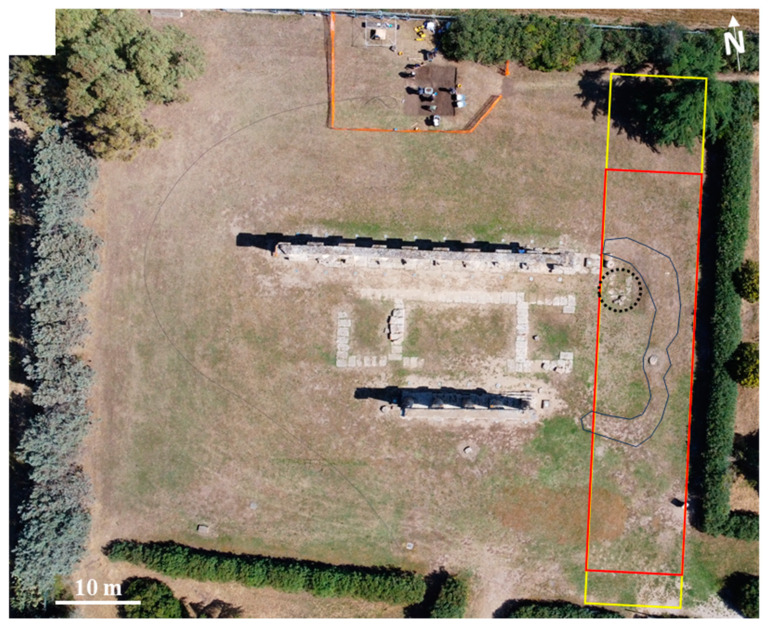
Archaeological site of Metaponto. Aerial image of the Hera temple with the yellow and red box indicating, respectively, the areas covered by ground and UAV magnetic datasets. The dotted circle highlights an outcropping calcarenite structure forming a relic at the base of the eastern temple side. The blue line encloses an area corresponding to a subtle change in vegetation, as discussed in the text.

Currently, on the northern side of the temple, there are 10 columns preserved out of the total 12, while only 6 columns remain on its southern side (Figure 1). On the smaller sides, the colonnade is not preserved at all. Therefore, in the easternmost part of the northern side of the temple, two columns were destroyed, and consequently, in origin, the northern colonnade extended toward the east for a further approximately 3 m, corresponding to the space relating to the two missing columns.

The eastern side, corresponding to the entrance to the temple, was investigated with our magnetic survey in search of buried structures. From other ground magnetic surveys in the area, a correlation was noted between magnetic lows and the presence of buried or sub-outcropping calcarenite walls.

### Drone-Borne and Ground Magnetic Surveys’ Design

**Figure 2:**
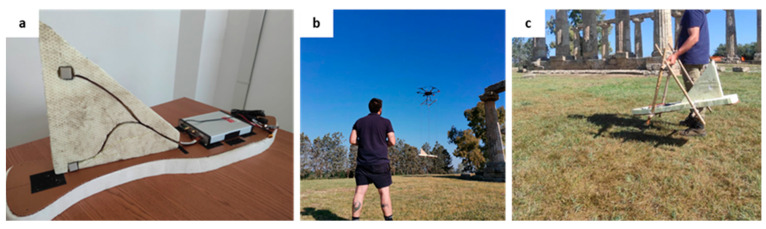
MFAM configurations used in the surveys. (**a**) Geometrics MFAM “Development kit” arrangement inside the prototype bird with the additional frame, which allows the sensors to be separated; (**b**) UAV flight configuration; (**c**) Ground configuration.

The magnetometer used for both ground and UAV surveys is the Geometrics Micro-Fabricated Atomic Magnetometer (MFAM) in the “Development kit” version, a Cs-vapor magnetometer widely used for UAV applications. In addition to its high sensitivity (1–5 pT/√Hz), wide dynamic range (20,000–100,000 nT), digital resolution of 0.05 pT/LSb (32-bit magnetometer output), small weight and volume, the main MFAM peculiarity is the high sampling frequency of 1000 Hz, which allows the correct sampling for the most obvious noise components, i.e., those generated by the drone and by power lines. MFAM has built-in IMU (inertial measurement unit) sensors to control flight stability by measuring the pitch, roll and yaw angles. The magnetometer was housed in the custom bird presented in previous papers by our research group ([14,16]). However, to perform the total field vertical gradient measurements, the light, aerodynamic, and nonmagnetic polystyrene bird with a thin and rigid base was modified with the addition of a fin-shaped polystyrene frame where the two sensors, at a distance of 0.25 m, are housed (Figure 2a). This small baseline for measuring gradients in archaeological research has been used in previous studies [23]. In that case ([23]), an elongated anomaly visible in the vertical gradient was confirmed to be related to archaeological remains by subsequent excavations, which brought to light a small road paved with fragments of baked bricks at about 0.5 m depth. In their study, Ref. [23] compared various baseline distances (0.25 m, 0.50 m, 0.75 m, and 1 m) and selected a 0.25 m sensor distance as the most successful in reducing noise generated by magnetic material dispersed on the ground. Moreover, theoretically, the vertical gradient is best approximated when the distance between the two sensors is small compared to the distance to the magnetized source (e.g., [31]).

The MFAM system incorporates an Adafruit GNSS user equipment module, which provides a positioning with an accuracy of 2–3 m. To enhance precision, a compact and cost-effective multi-GNSS receiver was added to our system (Emlid Reach M+) and located in our prototype bird, which also contains the magnetometer sensors. We employed the Reach M+ without a second receiver serving as a base station. Consequently, our operations were conducted in the PPK mode. For post-processing, we accessed base station data from Regional GNSS networks, specifically Permanent GPS and GLONASS stations. With the post-processing of positioning data (PPK mode), the Reach M+ can deliver coordinates that are accurate to centimeters.

The choice of the area covered by the UAV magnetic survey was conditioned by the presence of obstacles, like tall trees and a hedge-delimiting part of the site. Usually, these problems can be overcome by flying over with drones, but for data quality reasons, we preferred to fly as close as possible to the ground. In previous works ([14,15,16]), we have shown that in the case of surveys over intense magnetic anomalies, a practical and safe solution to carry the magnetometer can be to attach it rigidly to the drone landing gear at a small distance from the engines. However, in this study, considering the low-amplitude magnetic fields expected from the archaeological targets, we preferred to suspend the magnetometer 3 m below the platform (Figure 2b), which is a distance widely used by many practitioners.

The idea of suspending the magnetometer 3 m away from the UAV platform was derived from several studies dealing with the problem of minimizing drone interference while maintaining a stable flight. In fact, when the magnetometer is a few meters from the drone, the magnetostatic and electromagnetic noise due to the drone decreases substantially, but the flight is characterized by wider oscillations of the system with a degradation of the data quality (e.g., [32]). In fact, the swinging of the suspended magnetometer within the high-gradient electromagnetic field produced by the multi-rotor UAV generates a periodic, high-frequency signal in the magnetic measurements. Ref. [33] suspended a GEM Systems GSMP-35A magnetometer 3 m below a multi-rotor. The same 3 m distance was also used by [8,32]. Ref. [18] used an MFAM magnetometer in the MagArrow version for archaeological purposes by attaching it to the UAV via 2.5 m long ropes. Ref. [34], after the measurements of the electromagnetic interference generated by two multi-rotor UAVs in a controlled laboratory setting, used a MagArrow 3 m from the platform engines, showing that adequate sensor placement and pre-flight evaluation of the platform-sensor interactions provide useful mitigation strategies for UAV magnetic interference. Ref. [14] used a 3 m distance and successfully detected buried metallic pipes and cables. Ref. [35] tested different suspension configurations, using both a scalar and a vector magnetometer at 2.8 m from the platform. In summary, a distance of 3 m for a suspended magnetometer appears to be the most common compromise choice for obtaining a good-quality magnetic drone-borne dataset.

Therefore, the altitude of the UAV flight was 7 m, and the surveyed area was 35 × 10 m^2^ (Figure 1). Considering the flight altitude and the fact that the magnetometer was hung to the UAV by four 3 m long ropes, the resulting altitude of the magnetic sensors above ground level was 4 m. Due to the 1000 Hz magnetometer sampling rate and the 2 m/s flight speed, magnetic data were measured every 2 mm along the 11 survey lines. The survey lines were flown in an approximately North–South direction, with a 1 m distance between them. To ensure a constant acquisition altitude above the terrain, the flight path was planned using a high-resolution DSM (1 m × 1 m) generated with photogrammetric data surveyed just before the magnetic survey. MFAM has a polar dead zone, meaning that little or no signal is generated if the optical axes of the sensors lie in a direction +/−35° from the direction of the local Earth’s magnetic vector. To solve this problem, we arranged our flight lines in an approximately North–South direction, with the optical axes of the sensors constantly oriented in a West–East direction.

We also performed a ground survey with the MFAM in the same gradiometric configuration. The area was slightly more extended in the North–South direction than that in the UAV survey (Figure 1). In this case, the sensor closer to the ground was carried at about 0.30 m from the ground (Figure 2c). The acquisition speed, and thus, the measurement spacing along the survey lines, was similar to that of the UAV survey. The line spacing was 0.50 m, and the survey lines were oriented the same as those in the UAV survey.

Overall, the duration of the flight was approximately less than 5 min, while the ground acquisition took more than 30 min.

**Figure 3:**
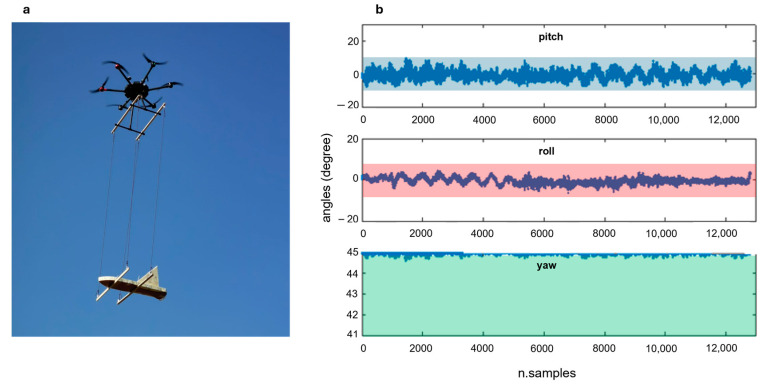
(**a**) To prevent strong oscillations of the magnetometer during flight, the anchor points of the support ropes were separated using wooden supports; (**b**) pitch, roll, and yaw data were collected during a flight under strong wind flight conditions. The blue, red, and green areas indicate the range limits recommended by Walter et al. (2019; ±5° yaw, ±10° pitch, and roll) [32].

In the case of wind (as it was at the time of the survey), the choice of suspending the magnetometer using 3 m long ropes can create unwanted oscillations in the sensors, which could compromise both the stability of the flight and the data quality. To overcome these problems, we improved the stability of the flight configuration by adding a wooden support that widened both the suspended magnetometer support base and the distance between anchor points at the base of the drone (Figure 3a). We checked the pitch, roll, and yaw data (Figure 3), representative of the three principal axes of rotation of the system during flight, collected along some test lines and observed that all the points fall in the magnetometer attitude variations recommended by [32] (±5° yaw, ±10° pitch, and roll).

Ref. [32] approximated the frequency of the periodic swinging of a suspended payload below a UAV to that of an imperfect pendulum. Considering typical cable lengths of 3 or 5 m, the estimated periodic swinging has a frequency from 0.4 to 0.2 Hz. However, the hung magnetometer is not a perfect pendulum oscillating in a two-dimensional space. Moreover, several external effects, like the strong swinging during the turns at the end of the flight profiles or windy conditions, may produce oscillations at different frequencies. These oscillations of the magnetometer can create variations in the magnetic field measurements generated by the gradients of the magnetic field in the volume where the sensors oscillate. To correctly predict the effect of these oscillations, we carried out a spectral analysis of the Total Magnetic Intensity (TMI) measurements acquired during some tests where we simulated a swinging motion of the MFAM with respect to the pitch axis at three different oscillation frequencies. The same test was repeated with respect to the roll axis by fixing the pitch and the yaw.

**Figure 4:**
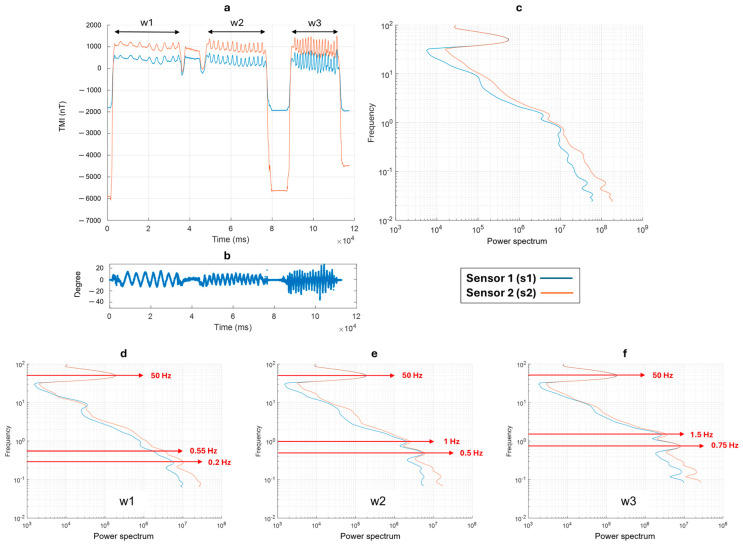
(**a**) Total magnetic intensity data acquired during the pitch swinging test; (**b**) Variation of the pitch during the test; (**c**) Power spectrum of the entire dataset; (**d**) Power spectrum for the data collected between the time range 2.5–37 s (w1); (**e**) Power spectrum for the data collected between the time range 47–77 s (w2); (**f**) Power spectrum for the data collected between the time range 89–112 s (w3).

Figure 4a shows the TMI variation in the magnetic data in the time domain during the first test (pitch). The orange (s2) and blue (s1) lines indicate, respectively, the signals of sensor 2 (the sensor closer to the ground) and sensor 1. We can distinguish three moments of different swinging motion frequency: (1) the time window 1 (w1) between 2.5 s and 37 s; (2) the time window 1 (w2) between 47 s and 77 s; (3) the time window 1 (w3) between 89 s and 112 s. The oscillation frequency increases from w1 to w3. The pitch data variations relative to the TMI measurements are shown in Figure 4b. During the highest frequency test (time window w3), we can see that the oscillations exceeded +/−20°. We obtained a scalogram of the datasets in the time domain by the continuous wavelet transform (CWT), using the “Morse wavelet” ([36]). From this scalogram, we obtained the power spectrum by summing the power of each single frequency at all times. We present the power spectrum in Figure 4c, which summarizes the spectral content of the entire dataset and for each time window (w1, w2, and w3), as shown in Figure 4d–f, respectively. Spectral analysis allows for the identification of several spectral contributions to the field. The prominent peak around 50 Hz is a common feature that can be interpreted as the contribution of the electrical power lines present in the area. The contributions due to the swinging motion are identified in w1 (Figure 4d) at 0.2 Hz (with a very small amplitude) and 0.55 Hz. As expected, in w2 and w3 (when the oscillation frequency increases), the two peaks are at higher frequencies, 0.5 Hz and 1 Hz, and 0.75 Hz and 1.5 Hz, respectively. We noticed that by increasing the oscillation frequency from w1 to w3, the amplitudes of the frequency peaks measured at the two magnetometer sensors (s1 and s2) became similar.

**Figure 5:**
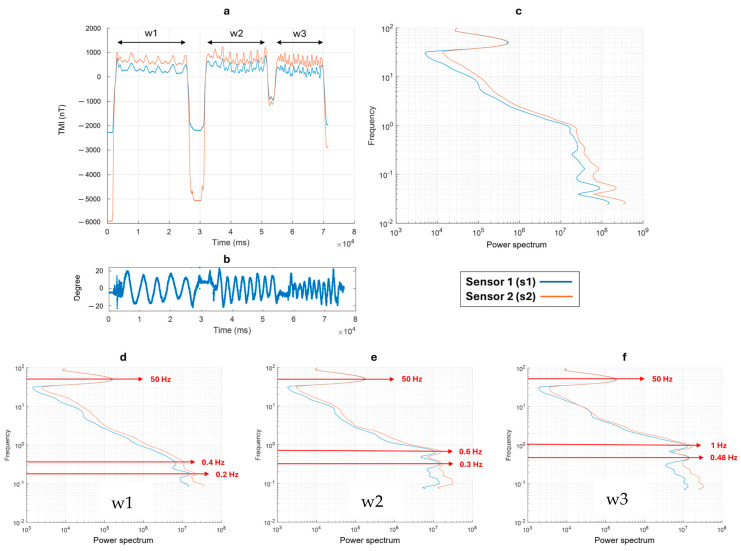
(**a**) Total magnetic intensity data acquired during the roll swinging test; (**b**) variation of the roll during the test; (**c**) Power spectrum of the entire dataset; (**d**) Power spectrum for the data collected between the time range 2.8–28 s (w1); (**e**) Power spectrum for the data collected between the time range 32–52 s (w2); (**f**) Power spectrum for the data collected between the time range 54–70 s (w3).

The second test, involving oscillations with respect to the roll axis (i.e., around the long axis of the magnetometer bird), showed a similar result. The variation in the magnetic data in the time domain is shown in Figure 5. Like the previous test, we varied the swinging frequency in three time windows, namely: (1) w1 between 2.8 s and 28 s; (2) w2 between 32 s and 52 s; (3) w3 between 54 s and 70 s. The relative roll variations (between +/−20°) and the spectral content of the entire dataset are shown, respectively, in Figure 5b,c. The spectral analyses relative to each time window are shown in Figure 5d–f. In w1 (Figure 5d), the frequency peaks due to the swinging motion are identified at 0.2 Hz and 0.4 Hz. At w2, these peaks are at 0.3 Hz and 0.6 Hz. Finally, in w3, the two peaks are at 0.48 Hz and 1 Hz.

Thus, both tests show that the spectral counterpart of the swinging motion along different axes is represented by two distinct peaks. Recognizing that the signal produced by the oscillations of the suspended magnetometer can be represented in the spectrum by peaks at more than one single frequency is important for identifying these signals in real data and for designing appropriate filters for their elimination.

## 3. Results

### 3.1. UAV Magnetic Survey

**Figure 6:**
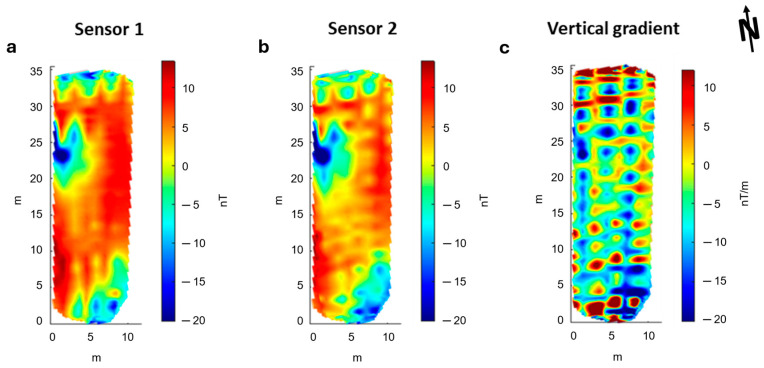
UAV Unfiltered Total-Field Anomaly (TFA) maps. (**a**) TFA map at sensor 1 (altitude 4.25 m agl); (**b**) TFA map at sensor 2 (altitude 4 m agl); (**c**) Vertical gradient map.

Figure 6 shows a map of the drone-borne Total-Field Anomaly (TFA) measured at both magnetometer sensors and the map of the relative vertical gradient. The measurements are unfiltered. The sensor closer to the ground (s2) was at 4 m elevation, and the other one (s1) was 0.25 m above. TFA maps were obtained after the removal of the data relative to the UAV turns between the survey lines and a constant value representing the IGRF (46,670.1 nT) in the area. The data obtained from both sensors (Figure 6a,b) are characterized by a strong heading error depending on the orientation of the sensors and caused by the bi-directional flight mode (South–North and North–South along adjacent lines). This error can be recognized from the different mean values of the field between the adjacent flight lines. Moreover, a strong wind during the acquisition caused oscillations in the suspended magnetometer, with effects more evident in the map of sensor 2 (Figure 6b). These effects are generated by the gradients of the magnetic field in the volume at which the sensors oscillate. This swinging characteristic of the acquired field strongly influences the computed vertical gradient (Figure 6c), which shows an alternation of magnetic highs and lows with a wavelength of about 2.5 m.

**Figure 7:**
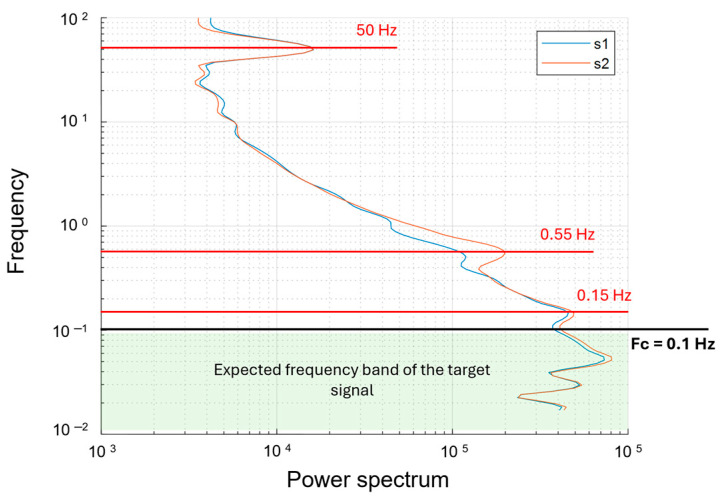
Power spectrum of UAV magnetic data. The blue and orange lines are referred to, respectively, the first and second sensor signals. The red lines indicate the mean noise peaks: (1) 50 Hz relative to the power lines; (2) 0.55 Hz and 0.15 Hz are interpreted as generated by the oscillations of the system. The cyan area, at frequencies lower than 0.1 Hz, highlights the frequency band associated with the target signal that will be used as the cut-off frequency for low-pass filtering of the noisy components at 50, 0.55 and 0.15 Hz.

We explored the spectral content of the acquired signal by an analysis of the time domain through the CWT. We show in Figure 7 the power spectrum for both sensors’ datasets. The most evident spectral peak is around 50 Hz, interpretable as due to the presence of alternate fields generated by the AC power lines present in the area and, to a much lesser extent, due to the magnetic and electromagnetic fields generated by the UAV platform ([34]). In fact, this peak has the same amplitude for both sensors despite their different distances from the drone. At lower frequencies, the peaks at 0.55 Hz and 0.15 Hz could be instead associated with the oscillation of the MFAM, consistently with the tests described in Figure 4 and Figure 5. To estimate the frequency band that can be roughly associated with the target signal, we use the same approach described in [14,34]. First, we select the anomaly of interest (the one we consider a useful signal). Then, we evaluate the ratio of the UAV speed (2 m/s) and the anomaly horizontal extent (representing a half-wavelength of the signal) multiplied by two. Here, the analysis of each profile shows a half-wavelength of 15–25 m for the anomaly of interest, which combined with a survey speed of 2 m/s, gives a target signal frequency band ranging between 0.06 and 0.05 Hz. Thus, it can be seen that the target signals should not spectrally overlap with the swinging spectral contents, and we can design a simple filter to isolate the useful signal.

Therefore, we used 0.1 Hz as a cut-off frequency for a time-domain Hanning-window low-pass filter aimed at preserving the frequency band associated with the source and removing the higher frequency noise, including the signal due to the magnetometer oscillations. Moreover, we attenuate the heading-error features by subtracting the mean value of the magnetic field for each profile and by applying a directional filter based on the discrete wavelet transform ([24,37]).

**Figure 8:**
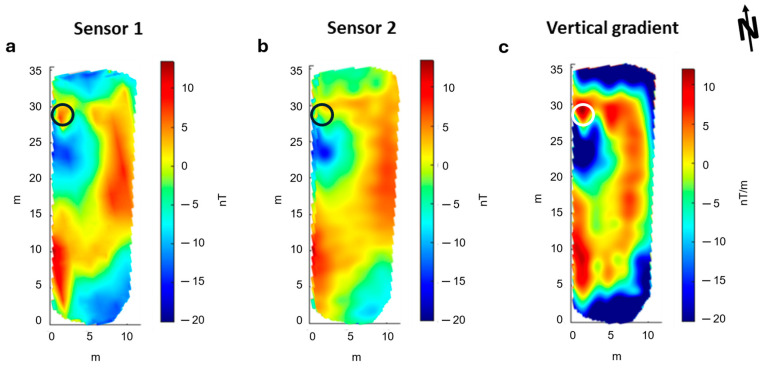
UAV filtered Total-Field Anomaly (TFA) maps. (**a**) TFA map at sensor 1 (altitude 4.25 m agl); (**b**) TFA map at sensor 2 (altitude 4 m agl); (**c**) Vertical gradient map. The small black or white circles mark the position of the spotlight (see text).

These processing steps allowed us to obtain the filtered maps shown in Figure 8. The TFA maps of both sensors display an amplitude variation of about 35 nT, ranging from about −20 to 15 nT. In the vertical gradient map, the strongest anomaly is due to the presence of a spotlight at x = 2 m and y = 28 m. We noticed an extended magnetic high with an amplitude of about 5 nT/m, trending NS for some tens of meters. A magnetic low area to the west of the above-mentioned magnetic high is also very evident. The vertical gradient, as expected, has a better resolution than the TFA maps, especially in the eastern sector of the area, where the boundaries of the anomaly are better defined.

### 3.2. Ground Magnetic Survey

**Figure 9:**
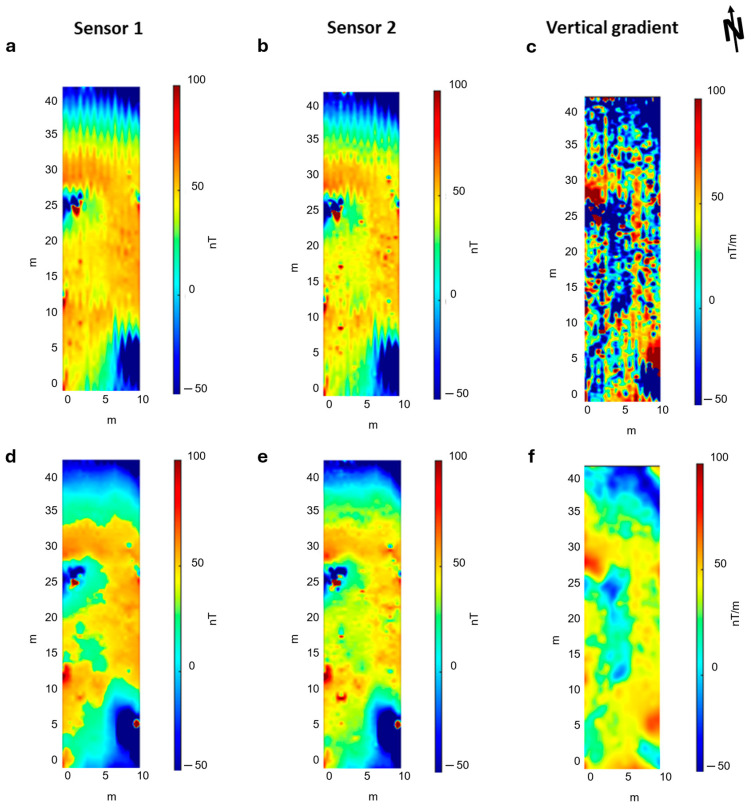
Ground magnetic maps. (**a**) Unfiltered TFA map obtained from sensor 1 (altitude 0.55 m agl); (**b**) Unfiltered TFA map obtained from sensor 2 (altitude 0.30 m agl); (**c**) Unfiltered Vertical Gradient map; (**d**) Filtered TFA map obtained from sensor 1 (altitude 0.55 m agl); (**e**) Filtered TFA map obtained from sensor 2 (altitude 0.30 m agl); (**f**) Filtered Vertical Gradient map.

**Figure 10:**
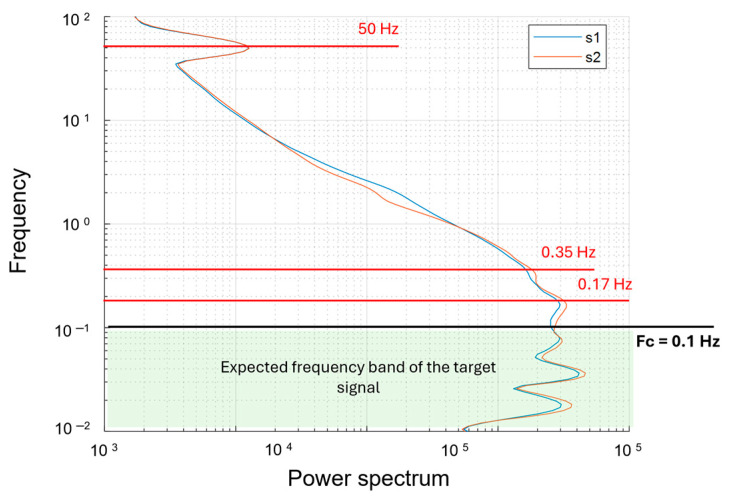
Power spectrum of the ground data. The blue and orange lines are referred to, respectively, the first and second sensor signals. The red lines indicate the mean noise peaks: (1) 50 Hz relative to the power lines; (2) 0.35 Hz and 0.17 Hz are due to the oscillation of the system. The cyan area is associated with the target signal at frequencies lower than 0.1 Hz that is used as the cut-off frequency for the low-pass filtering of the dataset.

The unfiltered magnetic maps relative to the ground survey (Figure 9) presents a better resolution than the UAV dataset. This is because, in this case, the survey lines were closer to each other and to the ground. The sensor closer to the ground (s2) was at an elevation of 0.30 m, and the other one (s1) was 0.25 m above. The spacing between the survey lines was 0.50 m. Similar to the drone-borne dataset, the unfiltered ground magnetic maps present a strong heading error, although they are relatively free from the noise due to the oscillation of the magnetometer (Figure 9a,b). This is clear in the vertical gradient result (Figure 9c), which reflects only the heading error effects, with alternating striped anomalies characterized by different mean-field values along the survey direction. However, some oscillation effects generated during the walk are probably present even in this ground dataset, although with smaller amplitudes than in the drone case. In Figure 10, we show the power spectrum for both sensors relative to the ground dataset. We associated the peaks at 0.35 Hz and 0.17 Hz with the oscillation of the MFAM. The spectral peak at 50 Hz, like in UAV data, is due to the presence of alternate fields generated by the AC power lines. Again, the frequency band associated with the target signal ranges between 0.06 and 0.05 Hz. Therefore, we filtered the ground magnetic dataset using the same cut-off frequency chosen in the drone-borne case. The results are shown in Figure 9d–f.

The ground magnetic dataset shows the main magnetic feature detected in the UAV data, i.e., the NS trending magnetic high and a magnetic low at its western side. However, in the ground case, the shape of the magnetic features is better defined and has higher amplitudes because of the lower acquisition altitude. The amplitude of the magnetic field is significantly influenced by the presence of the spotlight (x = 2 m, y = 28 m), with a total amplitude variation of about 150 nT, ranging from about −50 to 100 nT. The vertical gradient map shows a better resolution of the N-S anomaly and its westward right-angle bend at its northern tip. In addition, the magnetic lows in the central part of the map are more evident than in the total field maps. Overall, the results obtained from ground and UAV surveys are very similar.

## 4. Discussion

**Figure 11:**
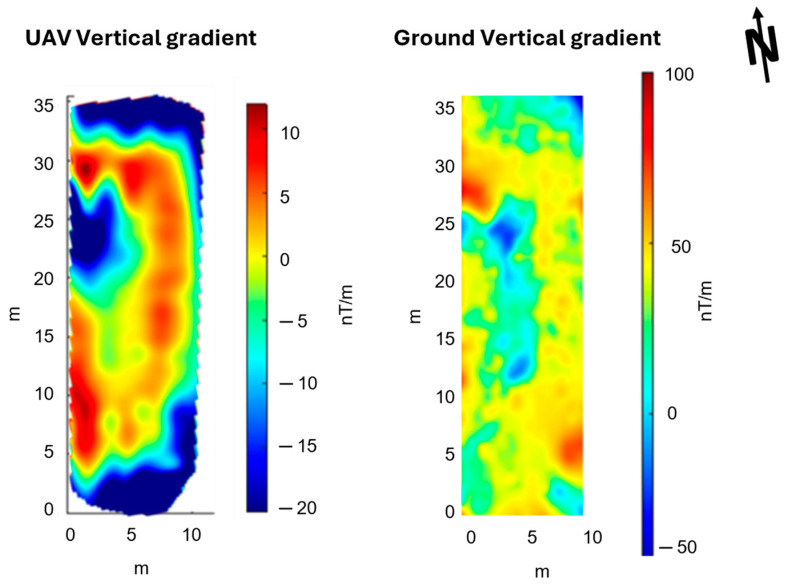
Side-by-side comparison of the vertical gradient maps obtained from drone-borne (on the **left**) and ground (on the **right**) datasets.

We showed that the vertical gradient data acquired by the UAV survey are fully compatible with those acquired during a ground-based survey (Figure 11). Both highlight an elongated NS trending magnetic high bounding from W, a magnetic low area. We notice that the northern part of the magnetic low corresponds to the outcropping calcarenitic structure interpretable as the foundation of the Eastern temple side (indicated by the dotted circle in Figure 1). This seems consistent with the mentioned correlation between calcarenitic walls and magnetic lows, which was noticed in magnetic ground datasets in this area. Thus, the magnetic low should mark the position of the eastern side of the temple. The magnetic high is strongly correlated to a subtle vegetational change with respect to the adjacent areas (indicated by a thin blue line in Figure 1). Such a change might be caused by a different quality of the soil, perhaps caused by a ditch successively re-filled. This warrants further investigation, which will be programmed to cover the entire area of the archaeological site.

The use of drones could help the magnetic investigation in archaeological areas, allowing surveys to extend over large areas or in sites of difficult ground access. In this study, we tested a vertical gradient configuration with an MFAM during both UAV and ground-based surveys. The two datasets were compared to evaluate the quality of the drone-borne vertical gradient and, thus, the feasibility of this kind of survey in archaeological applications. The results of this work suggest that with a suitable gradient configuration system and processing procedure the use of drones can have a positive impact on the use of magnetic surveys for archaeological purposes.

However, the flight configuration, the integration between the magnetometer and drone and the processing workflow should be carefully chosen. Perhaps the most important choice for having useful gradiometric data is the distance between the two sensors. We used a distance between the MFAM sensors of 0.25 m. This choice worked well in our case, perhaps because the target investigated was very shallow. However, the baseline should be increased in the case of deep targets or for UAV magnetic surveys at higher altitudes. In fact, we also have another gradient configuration in use, adapting our custom bird to allow a baseline of 1 m.

In this research, considering the archaeological context and the expected weak magnetic anomalies, we kept the magnetometer sensors outside the region of the highest drone interference by suspending the magnetometer at 3 m below the drone to improve the signal-to-noise ratio. However, this can compromise the stability of the MFAM during flight. The flight configuration proposed in this work (with the wooden supports that widen to 90 cm the distance between the anchor points at both the suspended magnetometer support base and drone landing gear; Figure 3a) successfully limited wide magnetometer swings, but in other cases, this improvement could be not enough to guarantee a clean signal.

Spectral analysis is an important tool for the detection of the signal frequency content generated by the magnetometer oscillation, as well as of the high-frequency noise due to power lines or the magnetic and electromagnetic interference of the drone. The spectral analysis is necessary to properly design a filter preserving the expected signal frequency band. To characterize the nature of the spectral features of the magnetic field due to the magnetometer oscillations, we conducted a practical test consisting of recording the magnetic field during magnetometer oscillations at different frequencies. Ref. [31] approximated the swinging frequency of a magnetometer in flight to that of an imperfect pendulum. However, our tests confirmed that the oscillation signals might be characterized by more than a single peak in the frequency domain. This result enabled a correct interpretation of the spectra and a proper filtering of our dataset.

## 5. Conclusions

In this work, we tested a vertical gradient configuration of an MFAM for archaeological purposes (both for drone and ground surveys). Results show that the measure of useful drone-based vertical gradients is possible, and we propose a specific flight configuration and processing workflow to mitigate noise and improve the data quality. Our final considerations encompass several crucial aspects. Firstly, a distance of 0.25 m between the MFAM sensors has proven effective for shallow targets at the altitude selected for the survey (sensors at 4 m from the ground). However, for high-altitude drone surveys or deeper targets, this distance should be increased. Secondly, a recommended practice for typical weak archaeological anomalies involves suspending the magnetometer 3 m below the drone. This strategic placement enhances the signal-to-noise ratio while minimizing the magnetic and electromagnetic interference caused by the aerial platform. Thirdly, we successfully implemented a flight configuration that introduced a wooden support, widening both the suspended magnetometer base and UAV support. This design mitigates wide magnetometer swings during flight. Additionally, our specifically designed test revealed that the noise caused by the sensors’ oscillation during flight could be characterized by a frequency content exhibiting even more than a single spectral peak. To isolate the useful signal, we applied a low-pass filter removing high-frequency noise, such as the one generated by oscillations of the suspended system, the magnetostatic and electromagnetic noise generated by the drone and the noise from power lines. Finally, a comparison of vertical gradient maps obtained from both UAV and ground-based datasets demonstrates good agreement, highlighting two magnetic anomalies, one of which is probably related to the temple’s structure. These results show that drone-borne magnetic gradiometry can have a role in archaeological exploration.

## Data Availability

The datasets presented in this article are not readily available because the data are part of an ongoing study.
